# Spatial Coupling Coordination Evaluation of Mixed Land Use and Urban Vitality in Major Cities in China

**DOI:** 10.3390/ijerph192315586

**Published:** 2022-11-24

**Authors:** Lijing Dong, Lingyu Zhang

**Affiliations:** School of Public Administration, Liaoning University, Shenyang 110136, China

**Keywords:** major cities in China, mixed land use, urban vitality, spatial coupling coordination evaluation, influencing factors

## Abstract

Based on the data from 35 major cities in China in 2020, this paper applies the Simpson’s diversity index, the entropy value method, and the coupling coordination degree model to comprehensively measure the coupling coordination level of mixed land use and urban vitality in major cities in China and further analyze their spatial distribution characteristics. In addition, this paper analyzes the factors affecting the spatial variation of the coupling coordination level with the help of the geographic probe model. The study finds that: (1) The overall level of coupling coordination between mixed land use and urban vitality is high in 35 major cities in China. There is no disorder between mixed land use and urban vitality. (2) In terms of the spatial distribution of the coupling coordination between mixed land use and urban vitality in 35 cities in China, five cities, namely Beijing, Shenzhen, Shanghai, Guangzhou, and Chengdu, have the highest level of coupling coordination between mixed land use and urban vitality, reaching “good coordination” with a discrete spatial distribution. Central cities such as Hangzhou and Nanjing have the second highest level of coupling coordination and are at the “intermediate coordinate” with a “strip-like distribution” in space. Twenty cities in the north and south have the lowest coupling coordination levels and are in the “primary coordination.” Among these twenty cities, seven cities in the south have a higher level of coupling coordination than thirteen cities in the north, with a spatial distribution of a “C” shape. The northern cities have the lowest level of coupling coordination, with a “W”-shaped distribution in space. (3) Population size plays an essential role in guiding the level of coupling coordination between mixed land use and urban vitality in major cities in China, followed by government regulation and economic level. At the same time, transportation conditions and industrial structure have the weakest influence on the level of coupling coordination between mixed land use and urban vitality in major cities in China.

## 1. Introduction

China’s urbanization rate has increased from 41.98% in 2010 to 63.89% in 2020. However, during the rapid urbanization process, the disorderly expansion of urban peripheries and inefficient land use in urban centers are common problems that severely constrain urban vitality. Subsequently, the National New Urbanization Plan (2014–2020) proposed to “strictly control the disorderly expansion of urban boundaries and promote the appropriate mix of urban land functions.” Moreover, the National Land Planning Outline (2016–2030) called for “comprehensively improve the level of economic and intensive land use, promote the multifunctional development of construction land, adjust and optimize the spatial structure of cities and towns, and strive to build a vibrant city.” This series of policies indicates that China’s urbanization is in a critical transition period from mid-term high-speed growth to late-stage quality improvement. China’s urban development mode is gradually shifting from extensible expansion to connotative growth and from the era of incremental volume to the age of stock. Based on international urban development experience, mixed land use can curb the ineffective expansion of urban boundaries, enhance the level of intensive urban land use, promote the multifunctional development of construction land, and stimulate urban vitality [1,2]. Therefore, quantitative identification of the coupling coordination between mixed land use and urban vitality in Chinese cities is vital for optimizing urban spatial layout, improving urban quality, and promoting high-quality urbanization development. 

Since the 1960s, scholars have been studying the relationship between mixed land use and urban vitality. Problems such as “separation of employment and residence,” influenced by the planning and construction concept of functional zoning, have restricted urban vitality. Based on this, Jacobs proposed in 1961 that mixed land use could effectively cope with the problems caused by functional zoning and stimulate urban vitality [1]. Mixed land use has both diversity and compatibility connotations. Diversity emphasizes the richness of land use types, and most scholars use the Simpson’s index, Shannon–Wiener index, entropy index, and dissimilarity index to calculate the degree of mixing [2,3,4,5]. Compatibility emphasizes the interrelationship between land use types [6], and academics mostly use the accessibility between land use types to evaluate the degree of mixing [7,8,9]. Studies on urban vitality are primarily based on two perspectives: urban sociology and urban morphology. Among them, the urban sociological perspective understands urban vitality as the reflection of the economy, society, and culture [10]. Urban morphology, on the other hand, considers urban vitality as urban life and activities shaped, and it was influenced by urban spatial form [11]. The macroscopic and microscopic perspectives of urban vitality have emerged based on the above two views. The macroscopic evaluation perspective of urban vitality primarily constructs the urban vitality evaluation index system from the dimensions of economic, social, cultural, ecological vitality, etc. It measures the vitality level of cities or regions at a large scale by using the fuzzy element model [12], random effect model [13], deviation maximization (DM) [14], etc. The microscopic evaluation of urban vitality uses geographic data such as cell phone base stations, location check-in data, Wi-Fi connection data, and points of interest to evaluate the vitality of streets and neighborhoods at small scales [15,16,17,18]. Studies on the association between mixed land use and urban vitality have concentrated on the impact of mixed land use on urban vitality [19], mainly in the United States [1], India [20], London [21], Canada [22], and other countries or cities. Studies show that mixed land use is positively associated with urban vitality [23,24,25] and that mixed land use enhances not only economic vitality and social equity [21,22] but also minimizes urban encroachment on the countryside and prevents urban sprawl [15]. In 2014, to promote nonmotorized transportation and create jobs, UN-Habitat required that 40% of the residential area be devoted to economic activities. It also adopted mixed land use as a fundamental principle of the New Strategy for Sustainable Community Planning, which states that mixed land use contributes to bridging economic disparities in communities [24]. Subsequently, policymakers and urban planners in Western Europe have proposed similar planning concepts, such as “urban intensification” and “compact cities,” which have produced similar positive results [24]. However, it has also been shown that mixed land use can lead to traffic congestion, social disorder, noise, high density of residential areas, and infrastructure stress, thus inhibiting urban vitality [26]. With advanced research, Ghosh (2022) showed that the impact of mixed land use on urban vitality has an inverted U-shaped trend. In other words, to a certain extent, mixed land use increases urban vitality, after which urban vitality decreases again as the degree of land mixed use increases [20]. We combed the existing literature and found abundant studies on mixed land use and urban vitality. The research on the connection between mixed land use and urban vitality has been intensifying. However, these studies are limited to one-way studies of the effects of mixed land use on urban vitality. With fewer two-way studies involving the relationship between the two, the research on the relationship between mixed land use and urban vitality is still in the initial stage. In addition, only some scholars have conducted relevant studies on individual cities in China, such as Nanjing [27], Chengdu [23], Shanghai [28], and Shenzhen [16], and breakdowns analyzing the relationship between mixed land use and urban vitality in Chinese cities based on a macro perspective are needed. 

In this paper, 35 major cities in China are used as the research objects to carry out the study. Firstly, the Simpson’s diversity index, entropy value method, and coupling coordination degree model are applied to measure the coupling coordination level between mixed land use and urban vitality in 35 major cities in China. Secondly, the spatial distribution characteristics of the coupling coordination level are analyzed with the help of the ArcGIS platform. Finally, the factors influencing the spatial variation of the coupling coordination level are investigated with the help of the geographic probe model. It is hoped that this study can deepen the knowledge of urban land use mix and urban vitality in 35 major cities in China, and provide a reference for urban planners to formulate urban planning policies, stimulate urban vitality, and generally grasp the overall situation of urbanization development in China.

## 2. Materials and Methods

### 2.1. Study Area

**Figure 1:**
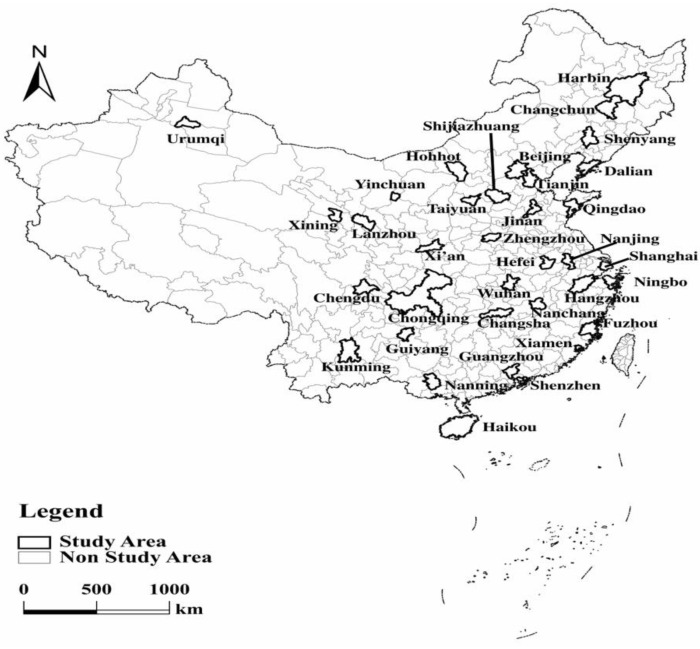
The distribution of 35 major cities in China.

In this paper, 35 major cities in China were selected as the study area (Figure 1). These cities include 4 municipalities directly under the central government, 5 planned cities, and 26 provincial capitals (Table 1). These 35 major cities, as the core cities of the region, generate 40.57% of China’s GDP in 2020 with a share of 28.03% of China’s population. As China’s urbanization development gradually shifts from the pursuit of speed to the pursuit of quality, urban development also shifts from epitaxial expansion to connotative growth. Cities are important carriers of people’s production and life. The mixed land use status of these major cities and the level of urban vitality are directly related to regional population changes, economic growth and urban planning, and even the overall situation of China’s urbanization.

### 2.2. Data Acquisition

Based on the principles of objectivity, scientificity, and accessibility, this study selects data on both urban land use and urban vitality in 2020 to analyze the level of coupling coordination between mixed land use and urban vitality in 35 major cities in China. The 2021 China Urban Construction Statistical Yearbook, the 2021 China Major Cities Commuting Monitoring Report, the 2021 China Urban Statistical Yearbook, the 2020 China Urban Vitality Research Report, the 2020 China Urban Transportation Report, and the Annual Report on Road Network Density in Major Chinese Cities of 2021 are this paper’s primary data sources. This study uses the corresponding data in 2019 to supplement individual missing data. In addition, the maps included in the text are based on the standard maps provided by the Standard Map Service website of the Map Technical Review Center of the Ministry of Natural Resources (Review No. GS(2020)4619), and the base map is unmodified.

### 2.3. Construction of Mixed Land Use and Urban Vitality Index System

Urban construction land is a general term for residential land, administration and public services land, commercial and business facilities, industrial land, logistics and warehouse land, road, street, and transportation land, municipal utilities land, and green space and squares. This paper uses urban construction land to characterize and measure the level of mixed land use, mainly with the following three considerations. (1) Normally, land can be classified into six types: arable land, forest land, grassland, water, urban and rural industrial, mining and residential land, and unused land. However, this classification standard is too macroscopic. The coupling coordination between mixed land use and urban vitality is not sufficiently detailed based on this classification standard, which is not enough for future urban planning work. (2) The classification statistics of urban construction land in China are based on the “Standard for Urban Land Classification and Planning of Construction Land GB50137” compiled by the Ministry of Housing and Urban-Rural Development of the People’s Republic of China. The classification standard is authoritative, objective, accurate, and fully aligned with China’s national conditions. (3) China’s urban construction land has the excellent municipal infrastructure and is the main area for residents to produce and live. Urban construction land is more closely related to urban vitality than other sites. Using it to characterize urban land and measure the extent of mixed land use is more in line with the purpose of this study.

Based on the specific indicators in the 2021 Annual Commuting Monitoring Report of Major Cities in China, the 2020 Annual China Urban Vitality Research Report, and the 2020 Annual China Urban Transportation Report, this paper refers to the urban vitality evaluation index dimensions constructed by Shi [10], Lan [13], and Lei [14] to construct the urban vitality evaluation index system of 35 major cities in China in four dimensions: economic, social, cultural, and ecological vitality (Table 2). Among them, economic vitality is the first driving force of urban development, characterized by the level of urban consumption and urban economic activity. The specific indicators used to measure it include retail sales of social consumer goods per capita and the nightlife index. Social vitality reflects the attractiveness of the city and the efficiency of urban operation. Its indicators include population attractiveness index, spatial radius of commuting, average one-way commuting distance, separation of employment and residence, peak congestion index of commuting, 45 min bus service capacity ratio, the proportion of commuting population within 5 km, and road network density. Cultural vitality is the soft power of a city, which measures the level of education, science and technology, and innovation in a city. Specific indicators include the proportion of education and science and technology expenditures in fiscal expenditures, the number of patents granted, the number of inventions, the number of museums, and the number of public library collections per capita. Ecological vitality is a measure of the city’s ecological environment and environmental protection level, with specific indicators including per capita green area, greening coverage rate of built-up areas, harmless treatment rate of domestic garbage, and centralized treatment rate of sewage treatment plants.

### 2.4. Mixed Land Use Measurement

Diversity is a vital arrow to measure the extent of mixed land use [9], and this paper introduces the ecological Simpson’s diversity index [29] to test the level of mixed land use. The formula is as follows:

(1) $$D_{i}=1-\sum_{l}^{t}P_{l}^{2},$$

where *D_i_* is the mixed land use index of the ith city; *P_l_* is the proportion of the area of the lth urban use type to the total size; *l* is the urban use type, and its maximum value *t* is 8. The value range of *D_i_* is [0,1], and a more significant value represents a more elevated level of urban mixed land use.

### 2.5. City Vitality Measure

- Entropy Value Method

To avoid the influence of subjective assignments on the results, this paper uses the entropy method to make the evaluation results more objective. The specific operation steps are as follows:

First, standardize the data. The polar difference method is applied to standardize the indicators to avoid the influence of the order of magnitude and unit differences between indicators. The indicators are divided into positive and negative indicators. The larger the positive indicator, the higher the level of the system, and the opposite for the negative indicator. The formula is as follows:

Positive indicators:

(2) $$X_{\mathrm{ij}}=\frac{Z_{\mathrm{ij}}{-\min(Z}_{\mathrm{ij}})}{{\max(Z}_{\mathrm{ij}}{)-\min(Z}_{\mathrm{ij}})}.$$

Negative indicators:

(3) $$X_{\mathrm{ij}}=\begin{array}{c} \frac{{\max(Z}_{\mathrm{ij}}{)-Z}_{\mathrm{ij}}}{{\max(Z}_{\mathrm{ij}}{)-\min(Z}_{\mathrm{ij}})} \end{array},$$

where *Z_ij_* is the original data, *X_ij_* is the normalized value, *i* (*i* = 1, 2,…, *m*) is the study region, and *j* (*j* = 1, 2,…, *n* ) is the study index. Since there are 0 values in the standardized data, which cannot be taken as logarithm in the subsequent calculation, the standardized values are added to 0.0001 and shifted in this paper.

Second, calculate the weight of indicator for the region:

(4) $$P_{\mathrm{ij}}{=X}_{\mathrm{ij}}\;/\;\sum_{i=1}^{m}X_{\mathrm{ij}}.$$

Third, calculate the entropy value:

(5) $$E_{j}=-t\sum_{i=1}^{m}P_{\mathrm{ij}}{\mathrm{lnP}}_{\mathrm{ij}},$$

where *t* = 1/*ln(m)*.

Fourth, calculate the coefficient:

(6) $$G_{j\;}{=1-E}_{j}.$$

Fifth, calculate the weight:

(7) $$W_{j}{=G}_{j}\;/\;\sum_{j=1}^{n}G_{j}.$$

- Linear Weighting Method

(8) $$S_{i}=\sum_{j=1}^{n}x_{\mathrm{ij}}W_{j},$$

where *S_i_* is the hybrid system index, and the level of system development increases with *S_i_*.

### 2.6. Coupling Coordination Degree Model

The coupling degree is a physical model to describe the degree of interaction and influence between or within elements of the system [30,31]. Since there is a strong interaction between mixed land use and urban vitality, the coupling degree in this paper can be used to study the interaction between mixed land use and urban vitality. In order to investigate the degree of association between mixed urban land use and urban vitality in major cities in China, the following coupling degree model is constructed:

(9) $$C=[S_{1}\times S_{2}∕{\;((S}_{1\;}+S_{2})\;∕{\;2)}^{2}{]}^{1/2}{=2(S}_{1}\times S_{2}{)}^{1/2}\;∕\;(S_{1}{+S}_{2}),$$

where *S*_1_ and *S*_2_ denote the levels of the two subsystems of mixed land use and urban vitality in major Chinese cities, respectively, and *C* means the subsystem coupling degree, with the value of *C* ranging from [0,1]. Considering that the coupling degree model can only represent the degree of association of subsystems and cannot determine how the level of coordination among subsystems, the coupling coordination degree model is introduced. The model is as follows:

(10) $${D=(C\times T)}^{1/2},$$

(11) $$\;T=\alpha S_{1\;}+\beta S_{2}.$$

In the formula, *D* is the coupling coordination degree, and the value range is [0,1]; *T* is the evaluation index of two subsystems; *α* and *β* are the contribution coefficients, *α* + *β* = 1. Since mixed land use is beneficial to enhance the level of urban vitality, and the level of urban vitality can optimize the mixed land use, the two are complementary systems, so let *α* = *β* = 0.5. In this paper, referring to the research of Liao [32], we use the uniform distribution function method to classify the level of coupling coordination between mixed land use and urban vitality, and the classification criteria are as follows (Table 3).

### 2.7. Geographic Probe Model

The geographic probe is a statistical method for detecting the spatial differentiation of something and revealing its drivers [33]. The geographic probe includes four significant parts: factor, interaction, risk, and ecological detection. The core idea of the factor detection module is that geographic things constantly exist in a specific spatial location, and the environmental factors that influence their development and change are spatially differentiated. If an environmental factor and the change of a geographic thing have significant spatial consistency, this environmental factor is decisive for the occurrence and development of a geographic thing [34]. Interaction detection can identify whether the influencing factors act independently or interactively. Expressly, interaction detection assumes that *F*_1_ and *F*_2_ are the two factors influencing the spatial differentiation of *W*. The new layer *F*_1_ ∩ *F*_2_ is formed by spatially superimposing *F*_1_ and *F*_2_, and *F*_1_ and *F*_2_ jointly determine the properties of the new layer. Firstly, we calculate the degree of influence of two factors, *F*_1_, *F*_2_, and the superimposed layer *F*_1_ ∩ *F*_2_ on *W* spatial differentiation, i.e., *q* (F_1_), *q* (*F*_2_), *q* (*F*_1_ ∩ *F*_2_). Secondly, we compare *q* (*F*_1_), *q* (*F*_2_), and *q* (*F*_1_ ∩ *F*_2_) to reveal the interaction between different factors *F_i_* on *W* spatial differentiation and assess whether this interaction enhances or weakens the degree of influence of a single factor *F_i_* on *W* spatial differentiation [35]. The “factor detection” assumes the existence of variables *W*, *F_i_* (*i* = 1,…, *I*) and compares whether the spatial divergence of *W* is significantly consistent with that of *F_i_*. If it is, it indicates that *F_i_* influences the spatial variation of *W*, and its degree is measured by *q*, as shown in Equation (12). In this paper, we mainly use factor and interaction probing to examine the drivers of the level of coupling coordination between mixed land use and urban vitality in major cities in China.

(12) $$D_{i}=1-\sum_{l}^{t}P_{l}^{2}q=1-\frac{1}{{n\sigma }^{2}}\sum_{h=1}^{L}N_{h}\sigma_{h}^{2},$$

where *q* denotes the role of factors affecting the spatial divergence of *W*. The values range from [0,1]. *q* = 0 indicates that *U_i_* does not influence the spatial divergence of *W*, and *q* = 1 indicates that *U_i_* controls the spatial distribution of *W*. *N* denotes the number of samples in all regions, *h* = 1,…, *L*, *h* denotes the partitions of *W* and *U_i_*. *N_h_* denotes the number of subregion *h* samples. It is important to emphasize that the *q* value is the spatial variance of the independent variable *F_i_* explaining the spatial variance of the dependent variable *W*. Even without the significance test for the *q* value, *q* still has clear physical significance and does not obey the normal distribution assumption. When using the geographic probe to reveal the spatial divergence drivers of something, the drivers need to be partitioned. This paper divides each indicator into eight hierarchical zones using the natural breakpoint method. The extent of influence of each factor on the spatial divergence of the level of coupling coordination between mixed land use and urban vitality is calculated separately by the geographic probe.

## 3. Results

### 3.1. Spatial Coupling Coordination Evaluation Based on the Coupling Coordination Degree Model

**Figure 2:**
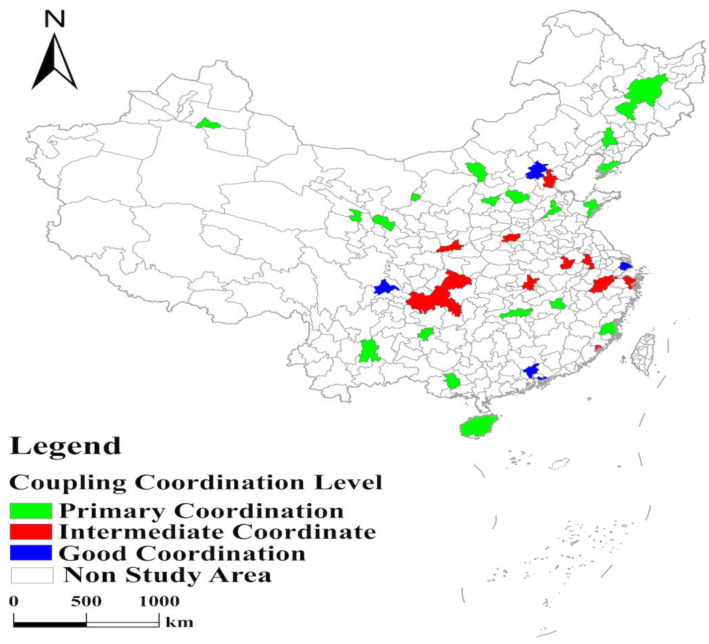
Spatial coupling coordination evaluation.

With the help of the coupling coordination degree model, the study calculates the coupling coordination degree of mixed land use and urban vitality in 35 major cities in China in 2020 (Table 4). The results were visually expressed with the help of ArcGIS software based on the classification (Table 3) (Figure 2) [36,37]. As shown in Figure 2, five cities, namely Beijing, Shenzhen, Shanghai, Guangzhou, and Chengdu, have the highest level of coupling coordination between mixed land use and urban vitality, all of which have good coordination and are spatially distributed in a discrete manner. Their coupling coordination levels are all in the range of 0.80–0.89. Central cities’ coupling coordination levels are all in the range of 0.70–0.79 and have the second highest level, with medium coordination and a “strip” distribution in space. The cities in the north and south have a lower level of coupling coordination that their coupling coordination levels are all in the range of 0.60–0.69 and are in primary coordination, with a “C” and “W” spatial distribution, respectively.

#### 3.1.1. The Level of Coupling Coordination of Beijing, Shenzhen, Shanghai, Guangzhou, and Chengdu Is “Good” and Shows “Discrete Distribution” in Space

**Figure 3:**
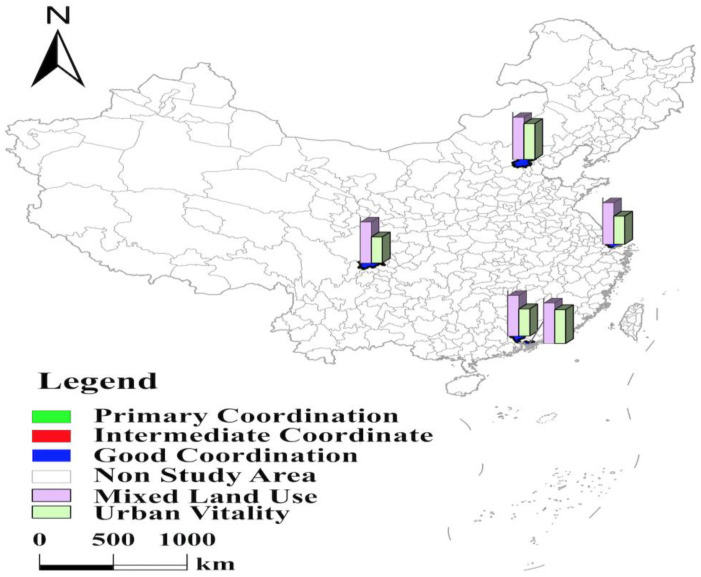
Levels of mixed land use, urban vitality, and coupling coordination in Beijing, Shenzhen, Shanghai, Guangzhou, and Chengdu.

Beijing, Shenzhen, Shanghai, Guangzhou, and Chengdu have the highest level of coupling coordination between mixed land use and urban vitality, with good coordination and discrete distribution in space (Figure 3). Beijing is the capital of China and the political and cultural center of China. Shenzhen is the first special economic zone and the window of China’s opening. Shanghai is China’s first coastal open city and is the economic center, trade center, and shipping center of China. Guangzhou is the national demonstration zone of independent innovation and a national demonstration zone for independent innovation and is known as the “southern gate” of China. Thanks to the solid economic foundation, superior geographical location, and the support of national policies, the four cities have a significant “siphon effect” on neighboring cities, attracting many industries, capital, population, and other factors to gather. They all have a resident population of over 17 million and a population attractiveness index of over 7.50, placing them in the top 4. At the same time, their nine indexes, such as the retail sales of social consumer goods per capita, the number of patents granted, and the harmless treatment rate of domestic garbage, exceed the average level of 35 cities. These advantages have laid a solid foundation for their economic, social, cultural, and ecological vitality. In addition, under the policy guidance of the Chinese government, the Beijing–Tianjin–Hebei city cluster, the Yangtze River Delta city cluster, and the Guangdong–Hong Kong–Macao Greater Bay Area have been formed with four cities as the core, respectively. Through population transfer, industrial optimization, and relocation, the industrial structure and the population layout of the four core cities of Beijing, Shenzhen, Shanghai, and Guangzhou have been continuously optimized, and the level of mixed land use has been continuously improved. The proportion of their residential land is 29.08%, 23.84%, 28.27%, and 30.91%, respectively, which is lower than the average of 35 cities and provides space for other types of construction land. Specifically, Shanghai ranks 1st in the proportion of municipal utilities land, Shenzhen ranks 2nd in the proportion of road, street, and transportation land, Guangzhou ranks 3rd in the proportion of administration and public services land, and Beijing ranks 7th in the proportion of administration and public services land.

Chengdu is the core city of the Chengdu–Chongqing city cluster and a critical node of “One Belt and One Road” and the development of the Yangtze River Economic Belt. On 2 October 2014, the State Council approved the “Application of the People’s Government of Sichuan Province on the Approval of Chengdu Tianfu New Area as a National New Area,” and Tianfu New Area was listed as a national new area. With the support of many national policies, Chengdu’s nightlife index, road network density, and more than ten other indicators are higher than the average of 35 cities. In particular, its number of public library collections per capita is even higher at 8.58, compared to Beijing’s 3.31. While receiving strong support from national policies, Chengdu is also actively exploring the road to sustainable development. In 2016, the Chengdu government proposed the plan of “Dual Core Co-prosperity, One City, Many Cities.” The project vigorously promotes the joint development of Tianfu New Area and the main urban area. At the same time, it actively promotes the close connection between the Chengdu metropolitan area and neighboring cities. Through continuous improvement of urban spatial planning, Chengdu has developed all kinds of urban construction land, so its value of mixed land use is at the forefront. With its high urban level and strong urban vitality, the degree of coupling coordination is tied with the four mega-cities of “Beijing, Shanghai, Guangzhou, and Shenzhen” and has achieved good coordination.

#### 3.1.2. The Level of Coupling Coordination of Central Part in 35 Major Cities Is at an “Intermediate Coordination” Level with a “Strip-like Distribution” in Space

**Figure 4:**
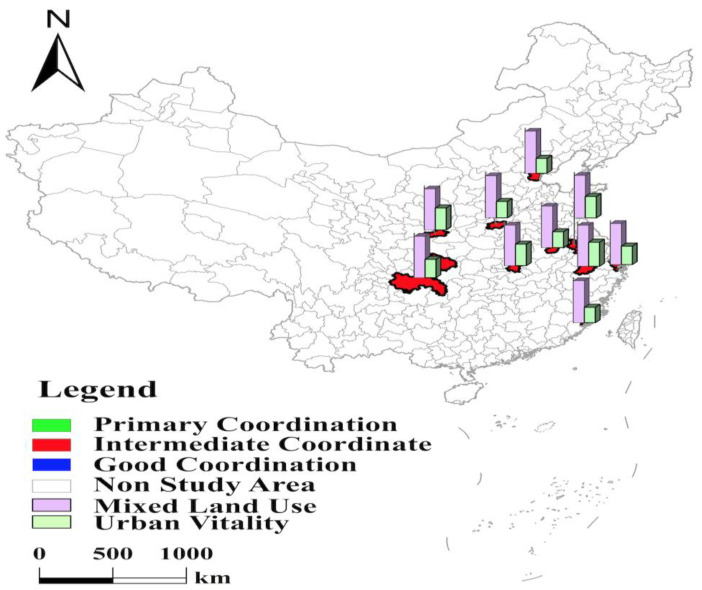
Levels of mixed land use, urban vitality, and coupling coordination in central part in 35 major cities.

Central cities such as Hangzhou are at the “intermediate coordination” level with a “strip-like distribution” in space. These cities are mainly located along the Yangtze River and are spatially distributed in strips (Figure 4). Among these ten cities, six are located in the Yangtze River Economic Belt, all are nodes of the “three growth poles” of the Yangtze River Economic Belt: Nanjing, Hangzhou, Hefei, and Ningbo are in the Yangtze River Delta city cluster, Wuhan is in the middle reaches of the Yangtze River city cluster, and Chongqing is the core city of the Chengdu–Chongqing city cluster. In September 2014, the government issued the “Guidance on Promoting the Development of Yangtze River Economic Belt by Relying on the Golden Waterway.” In May 2016, the “Outline of Yangtze River Economic Belt Development Plan” was officially issued. With the implementation of policies, the Yangtze River Economic Belt is moving toward the spatial pattern of “one axis, two wings, three poles, and multiple points.” Compared with the higher level of mixed land use, their urban vitality, especially ecological vitality, needs further improvement. The Yangtze River Economic Belt Development Plan outlines that “protecting and restoring the ecological environment of the Yangtze River” is integral to developing the Yangtze River coastal economic belt. The cities along the river have actively carried out the shutdown, transformation, relocation, or conversion of chemical enterprises along the river, but there are still shortcomings in the ecological environment. Specifically, Hangzhou’s green space per capita is only 12.27, ranking at 25; Ningbo, Wuhan, and Nanjing’s centralized treatment rates of sewage treatment plants rank 33rd, 34th, and 35th, respectively; Chongqing’s harmless treatment rate of domestic garbage is only 93.84%, which is at the lowest level. Regardless, it is worth noting that the Yangtze River basin, as the cultural cradle of the Chinese nation, has high regional cultural vitality. These six cities are all in the top ten regarding the number of museums. Wuhan ranks 2nd in terms of the number of inventions, and Hefei has the most significant proportion of education and science and technology expenditures in fiscal expenditures. Thus, increasing innovation support, guiding the production sector to technological innovation, and growing the clean energy industry are feasible paths to enhance the level of coupling coordination.

The coupling coordination levels of four cities, Xi’an, Zhengzhou, Tianjin, and Xiamen, are similarly limited by the level of urban vitality (Figure 4). With these four cities as the core, Guanzhong city cluster, Zhongyuan city cluster, Beijing–Tianjin–Hebei city cluster, and Haixi Economic Belt are formed. The rapid development of urbanization, mainly in urban clusters, has activated the land market of small and medium-sized cities outside the core cities. These four core cities have gradually improved their urban functions through industrial optimization and population out-migration and with a high level of mixed land use. Especially Zhengzhou, the core city of the Central Plains city cluster, is actively developing high-tech and modern service industries, spreading and transferring traditional industries to surrounding satellite cities. It is in the top 10 of 35 cities in terms of the proportion of five land types: administration and public services land, logistics and warehouse land, road, street, and transportation land, municipal utilities land, and green space and square land, and a modern metropolis integrating administration, commerce, transportation, and tourism services is taking shape. However, these four cities’ lack of social vitality prevents the coupling coordination level from improving. In the context of synergistic regional development, cities are increasingly connected, and transportation level becomes an important indicator of city efficiency. Xi’an, Zhengzhou, and Tianjin all have a resident population of over 12 million, and the urban traffic load is serious. The three cities generally suffer from long commuting times and severe separation of jobs and residences. In addition, Xiamen faces the problem of insufficient population attractiveness due to high housing prices and the low level of development of high-tech industries. These problems affect the social vitality of the four cities and are not conducive to further improving the coupling coordination level.

#### 3.1.3. The Coupling Coordination Level in the North and South in 35 Major Cities in China Is at the “Primary Coordination” Level with “W”-Shaped and “C”-Shaped Distribution in Space, Respectively

**Figure 5:**
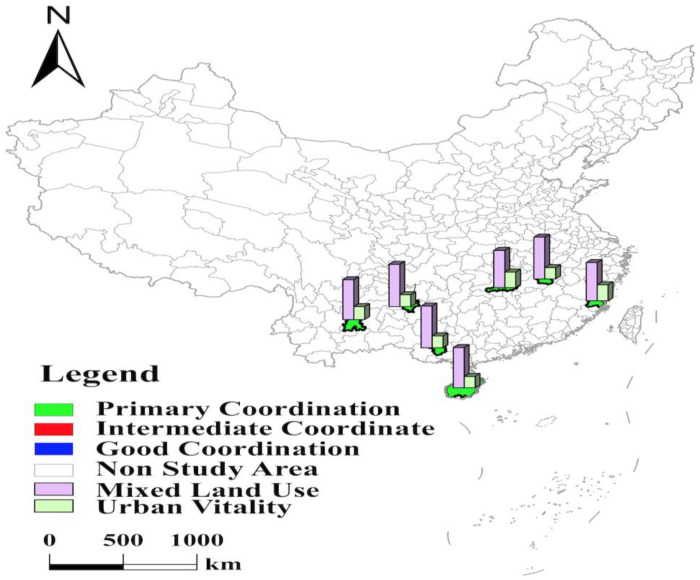
Levels of mixed land use, urban vitality, and coupling coordination in southern part in 35 major cities.

**Figure 6:**
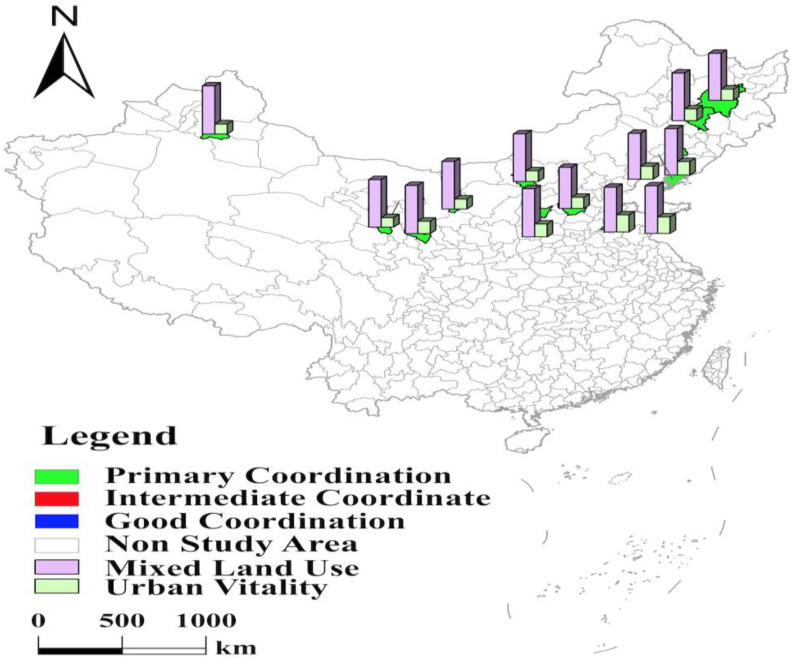
Levels of mixed land use, urban vitality, and coupling coordination in northern part in 35 major cities.

The cities with primary coordination include 20 cities, such as Qingdao and Fuzhou. Among them, seven cities, including Fuzhou, Haikou, Changsha, Kunming, Guiyang, Nanchang, and Nanning, are located in the southern part, with a spatial distribution with a “C” shape and an average coupling coordination level of 0.66 (Figure 5). The remaining thirteen cities are located in northern China, and their coupling coordination level is 0.63, with a spatial distribution of a “W” shape (Figure 6). The coupling coordination level of the southern cities is slightly higher than that of the northern cities. Among these seven cities in the south, Fuzhou, Changsha, Kunming, and Haikou have low mixed land use and urban vitality levels, constraining the coupling coordination level. Haikou’s coupling coordination level is shallow, with a high tertiary industry share of 80.49% and a high concentration of population, resulting in a high proportion of residential land. In addition, Haikou has been actively building Hainan Free Trade Port, and the proportion of road, street, and transportation land is as high as 22.32%. The proportion of residential land and road, street, and transportation land is too high to occupy the space of other types of construction land. So, the proportion of industrial land and public facilities land ranked 34th and 35th, respectively. In addition, Haikou has a small number of higher education institutions, insufficient local talents, and four cultural vitality indicators, such as the proportion of education and science and technology expenditures in fiscal expenditures, ranked in the bottom 10. The lack of cultural vitality directly affects its level of coupling coordination. The resident populations of Fuzhou, Changsha, and Kunming are 8.32 million, 10.06 million, and 8.46 million, respectively, and the large population size causes the residential land area to reach 39.26%, 46.41%, and 41.74%, respectively, which affects the level of mixed land use. Although Fuzhou is the capital of Fujian Province, the city’s first place is not high. Its competitive advantages, such as talent, are not significant enough compared with Xiamen and Quanzhou, and the number of patents granted and inventions do not reach the average level of 35 cities, resulting in low cultural vitality. Kunming’s industrial structure relies on resource-based and high-energy-consuming industries such as tobacco, minerals, and metallurgy, ignoring the innovation drive. The number of public library collections per capita is only 0.42, which is less than 1/8 of Beijing’s. Changsha has paid much attention to the innovation drive in recent years, vigorously developing new industries such as intelligent Internet vehicles and new-generation semiconductors. So, its cultural vitality has been improved. However, the ecological benefits have been neglected in the development process. The green space per capita is only 11.65 square meters, ranking 31st, which restricts the further improvement of the coupling coordination level. Three cities, Guiyang, Nanchang, and Nanning, pay insufficient attention to innovation development, resulting in low cultural vitality, making it difficult to obtain further improvement in the level of coupling coordination. Their four cultural vitality indicators are lower than the average level of 35 cities, and the number of inventions ranks 29th, 28th, and 27th, respectively. It is worth noting that Nanning benefits from its superior geographical location near the Guangdong–Hong Kong–Macao Greater Bay Area and ranks first among the 35 cities in the nightlife index. Nanning should integrate more actively into it to further improve coupling coordination.

The 13 cities in the north, such as Urumqi and Shijiazhuang, have a lower level of coupling coordination than those in the south. However, they are still at the primary coordination level and have a “W”-shaped distribution in space (Figure 6). These 13 cities have different levels of economic development, geographical location, and other factors, which lead to a low level of coupling coordination. Qingdao and Jinan are the “twin cores” of economic and social development in Shandong Province. The two cities are suffering from the siphoning of large city clusters such as Beijing–Tianjin–Hebei and Yangtze River Delta, and talents and industries are gradually moving to vital regions, with population attractiveness indexes of 2.62 and 2.59, respectively, which are less than 1/3 of Shenzhen’s. In addition, Qingdao and Jinan have similar and misaligned industries, and internal competition makes it difficult to release the economic vitality of the two cities. They rank 30th and 27th out of 35 cities in the nightlife index. They have gaps in development with regions such as Zhejiang and Guangdong, which are located on the coast and have a dual-core development. Urban vitality constrained the level of coupling coordination between Qingdao and Jinan. In addition, Shenyang, Dalian, Changchun, and Harbin are subprovincial cities in three northeastern provinces. With the support of several rounds of the “northeast revitalization” strategy, the economies of the four cities are in a strategic adjustment stage of climbing over the hurdles and carrying heavy loads. Their nightlife index ranked 33rd, 35th, 31st, and 32nd, respectively, and their retail sales of social consumer goods per capita ranked 16th, 31st, 34th, and 33rd, respectively. Coupled with the severe aging of the population and the enormous burden of social security expenditures such as pensions in the three northeastern provinces, their expenditures on education and science and technology account for 13.32%, 14.21%, 15.76%, and 11.2% of fiscal expenditures, respectively, and that of Beijing is as high as 21.77%. The low economic and cultural vitality constrains the vitality level of the four cities and directly affects the level of coupling coordination. Taiyuan’s economic development is dominated by heavy industries such as iron, steel, coal, and chemicals. The city lacks the support of strategic emerging pillar industries for its development, resulting in its insufficient ability to absorb employment and support the upgrading of traditional manufacturing industries. Its number of patents granted and inventions are less than 1/10 of Beijing’s, its population attractiveness index is only 2.01, ranking 27th, and its nightlife index and retail sales of social consumer goods per capita are lower than the average of 35 cities. Hohhot is the capital of the Inner Mongolia Autonomous Region, and the “Hohhot–Baotou–Erdos cluster” is the region’s development locomotive. However, the industrial chain of the three cities, in recent years, is not highly coordinated, and the transformation and upgrading of resource-based industries have been complex. There is a certain degree of low-level homogeneous competition between the three cities, and the advantageous position of Hohhot as the provincial capital is gradually declining. Hohhot’s two economic vitality indicators such as the population attractiveness index, four social vitality indicators such as the population attractiveness index, and five cultural vitality indicators, such as the proportion of education and science and technology expenditure to fiscal expenditure, are all lower than the average level of 35 cities. The urban vitality ranks 30th, directly affecting the coupling coordination level. Urumqi, Xining, Lanzhou, and Yinchuan are deeply inland in China and located in the northwest of the country. Their economic structures, mainly resource-based industries and traditional agriculture, make them insufficiently innovative, and cultural vitality indicators such as the number of patents granted and inventions are at the lowest level, with the weakest cultural vitality. Yinchuan and Xining have particularly low cultural vitality indicators, with the two cities ranking in the bottom 10 for five cultural vitality indicators, including the proportion of education and science and technology expenditures to fiscal expenditures. Shijiazhuang has the lower level of coupling coordination with urban vitality. Its urban construction land area is only 186.84 square kilometers, but its resident population is over 11 million. Shijiazhuang has a limited area of urban building land and a great contradiction between people and land, and the proportion of residential land is as high as 50.25%, which dramatically reduces the space for other types of building land, resulting in the lowest level of mixed land use among the 35 cities. In addition, although Shijiazhuang is the capital of Hebei Province, it is not deeply integrated into the Beijing–Tianjin–Hebei city cluster due to its southern location. It is ranked 29th and 35th in the nightlife index and retail sales of social consumer goods per capita, respectively. The economic vitality is too low, making Shijiazhuang’s city vitality in 30th place.

### 3.2. Detection of Influencing Factors Based on Geographic Probe Model

Following the principles of objectivity, typicality, and data availability, a total of five indicators were selected as the influencing factors of the spatial variation of the level of coupling coordination between mixed land use and urban vitality by using a combination of literature analysis [38,39,40,41,42,43,44,45] and expert consultation methods (Table 5). (1) Economic level: economic development determines the concentration of various city resources and factors, which profoundly affects the level of mixed land use and urban vitality, characterized by GDP per capita. (2) Industrial structure: cities are the carriers of tertiary industry concentration. The reasonable layout of the industrial structure enables each region to fully utilize its resources and location advantages to improve urban spatial planning and enhance urban vitality. It is characterized by the value added by the tertiary industry as a proportion of GDP. (3) Government regulation: as an “invisible hand,” the government influences the level of mixed land use and urban vitality through territorial and spatial planning, industrial structure optimization, and population policy adjustment, which is characterized by general public budget expenditure per capita. (4) Traffic conditions: traffic conditions directly reflect urban operation efficiency. Generally, the better the level of mixed land use, the better the urban transportation, and the more perfectly transportation facilities can stimulate the spatial spillover effect of production factors and stimulate urban vitality, expressed by the urban road area per capita. (5) Population size: people are the creators and participants of social activities. The larger the population size, the higher the need for mixed use of urban land and the higher the urban vitality, characterized by the number of residents.

#### 3.2.1. Dominant Factor Detection

The independent variables were logarithmized to eliminate heteroskedasticity’s effect, ensuring the data’s stability. With the level of coupling coordination as the dependent variable and the logarithms of the five factors screened above as the independent variables (Table 5), the geographic probe model was used to identify the dominant drivers of spatial differences in the coupling coordination of mixed land use and urban vitality in major Chinese cities. First, the raster data were extracted to points using the fishnet creation function in ArcGIS software. The natural breakpoint method (Jenks) transformed the independent variables from numerical to typological quantities. Secondly, the ArcGIS platform spatially matched the coupling coordination level with each influence factor. The factor influence coefficients of the coupling coordination level of mixed land use and urban vitality in major Chinese cities were measured (Table 6).

Based on the results, it can be seen that all factors passed the significance P-value test. The government regulation factor (X3) and the population size factor (X5) are higher than other factors, both exceeding 0.50, indicating that government regulation and population size have important guiding roles in promoting the level of coupling coordination between mixed land use and urban vitality. The explanatory power of the economic level factor (X1) is also more robust than the other factors, reaching 0.48, indicating that the importance of economic development level to the level of coupling coordination between mixed land use and urban vitality cannot be ignored. The explanatory power of the transportation condition factor (X4) is 0.33, which is relatively weak for the coupling coordination between mixed land use and urban vitality. The industrial structure factor (X2) has the weakest influence on the level of coupling coordination between mixed land use and urban vitality, with only 0.14.

#### 3.2.2. Dual-Factor Detection

The interaction of multiple influencing factors was further analyzed with the help of geographic probe interaction detection. From the analysis results (Table 7), the explanatory power of any dual-factor interaction is greater than that of a single factor. The types of dual-factor detection effects are all enhanced, and there are no independent and weakened types. This indicates that the joint action of both factors significantly increases the explanatory power of the coupling coordination level with urban vitality. Among them, the interactions of industrial structure ∩ transportation condition (X2 ∩ X4), government regulation ∩ population size (X3 ∩ X5), and transportation condition ∩ population size (X4 ∩ X5) all have explanatory power exceeding 0.90 on the spatial distribution characteristics of the level of coupling coordination.

The types of factor interactions are divided into two categories: dual-factor enhancement and nonlinear enhancement. Dual-factor enhancement means that the explanatory power after the interaction is greater than that of the factor with the most significant explanatory power among the two. Linear enhancement is when the explanatory power after the dual-factor interaction is greater than the sum of the explanatory power of the single factor. According to the interaction results (Table 7), among the ten sets of interaction detection results generated by the interaction of five elements, Linear enhancement involves economic level ∩ industrial structure (X1 ∩ X2), economic level ∩ transportation condition (X1 ∩ X4), industrial structure ∩ government regulation (X2 ∩ X3), industrial structure ∩ transportation condition (X2 ∩ X4), industrial structure ∩ population size (X2 ∩ X5), transportation condition ∩ population size (X4 ∩ X5). The effect of these six sets of interactions on the level of coupling coordination achieves an interaction that is greater than the sum of the effects of the single factors. In particular, the industrial structure factor has the weakest influence on the coupling coordination extent from the dominant factor detection result, which is only 0.14. However, among the six groups of interaction detection, it indicates that the industrial structure can significantly influence the coupling coordination level in regions with high economic development, good transportation conditions, reasonable government regulation, and a large population size. That should draw the attention of policymakers.

## 4. Conclusions

(1) The level of coupling coordination between mixed land use and urban vitality in 35 major cities in China can be classified as “good coordination,” “intermediate coordinated,” and “primary coordination.” The overall level of coupling coordination is high and does not appear dysfunctional.
(2) In 35 major cities in China, the level of coupling coordination between mixed land use and urban vitality in China shows the spatial characteristics of Beijing, Shenzhen, Shanghai, Guangzhou, and Chengdu being highest, central higher, north and south lowest (Figure 2, Table 4). Specifically, among these 35 cities, five cities, namely Beijing, Shenzhen, Shanghai, Guangzhou, and Chengdu, have the highest level of coupling coordination between mixed land use and urban vitality, reaching “good coordination” with a discrete spatial distribution. Central cities such as Hangzhou have the second highest level of coupling coordination and are at the “intermediate coordination” level with a “strip-like distribution” in space. The north and south cities have the lowest coupling coordination levels and are at the “primary coordination” level. Among these 20 cities, 7 cities in the south have a higher level of coupling coordination than 13 cities in the north, with a spatial distribution of a “C” shape. The northern cities have the lowest level of coupling coordination, with a “W”-shaped distribution in space.
(3) From the detection results of the influencing factors (Table 6 and Table 7), government regulation and population size extremely influence the level of coupling coordination between mixed land use and urban vitality in major cities in China. The economic level has a third significant influence, followed by transportation conditions. Industrial structure has a minor influence on the level of coupling coordination. In addition, compared with the single factor, the dual-factor interaction significantly influences the level of coupling coordination. Among them, the interactions between industrial structure and transportation conditions, government regulation and population size, and transportation conditions and population size are the strongest.

## 5. Discussion

### 5.1. Policy Implications

#### 5.1.1. Play the Role of the Central Cities as a Bridge between East and West, Spanning North and South

The central cities (10 cities with intermediate coordination) among 35 major cities in China are geographically located in the east and west, running north and south, and we should fully use them. The study results show that, except for Beijing, Shenzhen, Shanghai, Guangzhou, and Chengdu, the central cities among 35 major cities in China have a high level of coupling coordination between mixed urban land use and urban vitality. The central cities among 35 major cities in China have become the important hub for narrowing the gap between north and south and east and west in China, especially when cities such as Beijing are more maturely developed and can hardly bear more population. It is possible to guide the population and industries to gather in the central cities among 35 major cities in China, to make the central cities the vital growth pole to promote the balanced development of national space and lead regional economic development. On the one hand, it should appropriately expand the supply of construction land in the central cities among 35 major cities in China, improve the intensity of utilization of the stock of construction land, and improve the infrastructure and public services in the central region. On the other hand, it should continuously enhance the central cities’ ability to undertake the eastern region’s industrial transfer and labor division, strengthen the region’s internal driving force, and enhance its urban vitality while radiating the northern, western, and southern regions.

#### 5.1.2. Formulate Appropriate Policies to Guide the Reasonable Movement of Population

We are guiding moderate population clustering through reasonable government regulation. Since China’s reform and opening up, industries and employment have been concentrated in the eastern coastal regions, which has led to the spatial dislocation of market consumption areas and resource-rich areas and large-scale cross-regional movement of products and labor. That will undoubtedly increase the pressure on cities in the eastern regions and the lack of land development and urban vitality enhancement in other regions. From the study results, population size and government regulation significantly impact the level of coupling coordination between mixed land use and urban vitality in 35 major cities. Therefore, the government should delineate the boundaries of urban spatial development control and scientifically guide the population and industries to gather in areas with more powerful resources and environmental carrying capacity. By further defining the scale, structure, layout, and timing of urban development and utilization, the degree of mixed land use and urban vitality will be improved.

### 5.2. Contribution to Research, Limitations, and Prospects for Future Studies

Based on the data on land use and urban vitality of major cities in China in 2020, this paper comprehensively measures the level of coupling coordination between mixed land use and urban vitality in 35 major cities in China on the basis of constructing the mixed land use index system and urban vitality index system with the help of the Simpson’s diversity index, entropy value method, and coupling coordination degree model. On this basis, the paper also applies the geographic probe model to detect the influencing factors of the spatial distribution of the coupling coordination of mixed land use and urban vitality in 35 major cities in China, which has certain theoretical and practical significance. The current research on mixed land use and urban vitality is mostly based on the microscopic perspective to study the specific situation of individual cities [16,17,28], but there are few studies on the coupled and coordinated relationship between the two based on the macroscopic perspective. In addition, China is in a critical period of urbanization quality improvement, and the 35 cities selected in this paper, as the core cities in the region, will directly influence the changes in population, industry, and factors in the region. Consequently, this paper analyzes the spatial distribution of the coupling coordination level of mixed land use and urban vitality in 35 major cities and analyzes the main factors affecting it, which provides a reference for policymakers and urban planners to coordinate the relationship between the two. In addition, this study can also provide an important reference for other developing countries to coordinate the relationship between the two.

Both mixed land use and urban vitality are complex dynamic systems. Mixed land use is a complex issue involving diversity and accessibility, and urban vitality needs to consider multiple dimensions, such as political, economic, cultural, and ecological. This paper analyzes the coupled and coordinated relationship between mixed land use and urban vitality in China from a macro perspective, using major Chinese cities as the research unit. However, limited by data availability, this study lacks relevant data for core cities in Taiwan Province and Tibet Autonomous Region of China, as well as Hong Kong and Macao. In addition, this paper analyzes the spatial evolution of the coupling coordination between mixed land use and urban vitality in major cities in China based on cross-sectional data from 2020. In a future study, we will increase the temporal dimension and analyze the spatial and temporal evolution of the level of coupling coordination between mixed land use and urban vitality in major cities in China by collecting relevant data for more years. Additionally, on this basis, we will further explore the influencing factors that affect the spatial and temporal evolution of mixed land use and urban vitality, which will provide a more comprehensive and reliable reference for urban planners.

## Figures and Tables

**Table 1:** The selected 35 major cities in China.

Type	City
Municipality	Beijing, Shanghai, Tianjin, Chongqing
Single-Level Plan City	Shenzhen, Xiamen, Qingdao, Dalian, Ningbo
Provincial Capital City	Guangzhou, Chengdu, Hangzhou, Nanjing, Xi’an, Wuhan, Zhengzhou, Hefei, Jinan, Changsha, Fuzhou, Kunming, Guiyang, Nanning, Shenyang, Taiyuan, Nanchang, Lanzhou, Haikou, Changchun, Harbin, Hohhot, Urumqi, Yinchuan, Shijiazhuang, Xining

**Table 2:** Urban vitality evaluation index.

System Name	Observation Dimension	Evaluating Indicator	Unit	Attribute
Urban Vitality	Economic Vitality	Retail sales of social consumer goods per capita ^1^	Yuan	+
		Nightlife index ^2^	/	+
	Social Vitality	Population attractiveness index ^3^	/	+
		Spatial radius of commuting ^4^	Kilometer	+
		Average one-way commuting distance ^4^	Kilometer	-
		Separation of employment and residence ^4^	Kilometer	-
		Peak congestion index of commuting ^5^	/	-
		45 min bus service capacity ratio ^4^	%	+
		Proportion of commuting population within 5 km ^4^	%	+
		Road network density ^6^	km/km^2^	+
	Cultural Vitality	Proportion of education and science and technology expenditures in fiscal expenditures ^1^	%	+
		Number of patents granted ^1^	Number	+
		Number of inventions ^1^	Number	+
		Number of museums ^1^	Number	+
		Number of public library collections per capita ^1^	Number	+
	Ecological Vitality	Green space per capita ^7^	Square kilometer	+
		Greening coverage of built-up areas ^7^	%	+
		Harmless treatment rate of domestic garbage ^7^	%	+
		Centralized treatment rate of sewage treatment plants ^7^	%	+

^1^ Data source: the 2021 China Urban Statistical Yearbook (accessed on 26 June 2022). ^2^ Data source: http://mapopen.cdn.bcebos.com/cms/report/2019niandu/ (accessed on 26 June 2022). ^3^ Data source: https://huiyan.baidu.com/cms/report/2020nianduchengshi/index.html (accessed on 26 June 2022). ^4^ Data source: https://jiaotong.baidu.com/cms/reports/traffic/2020annualtrafficreport/index.html (accessed on 26 June 2022). ^5^ Data source: https://mp.weixin.qq.com/s/UB8AtxPfPq5s5ahu9f-65g (accessed on 26 June 2022). ^6^ Data source: https://www.caupd.com/think/zixun/detail/786.html (accessed on 26 June 2022). ^7^ Data source: the 2021 China Urban Construction Statistical Yearbook (accessed on 26 June 2022).

**Table 3:** Classification criteria for the level of coupling coordination between mixed land use and urban vitality.

Coupling Coordination Type	Coupling Coordination States
Serious imbalance	0.00~0.09
Severe disorders	0.10~0.19
Moderate disorders	0.20~0.29
Mild disorders	0.30~0.39
On the verge of disorder	0.40~0.49
On the verge of disorder barely coordinated	0.50~0.59
Primary coordination	0.60~0.69
Intermediate coordination	0.70~0.79
Good coordination	0.80~0.89
Perfect coordination	0.90~1.00

**Table 4:** Results of coupling coordination evaluation.

City	The Level of Mixed Land Use	The Level of Urban Vitality	The Level of Coupling Coordination	Coupling Coordination Type
Beijing	0.82	0.70	0.87	Good coordination
Shenzhen	0.79	0.66	0.85	
Shanghai	0.81	0.55	0.82	
Guangzhou	0.79	0.53	0.80	
Chengdu	0.80	0.51	0.80	
Hangzhou	0.80	0.47	0.78	Intermediate coordination
Nanjing	0.83	0.42	0.77	
Xi’an	0.80	0.43	0.77	
Wuhan	0.79	0.42	0.76	
Chongqing	0.81	0.36	0.73	
Ningbo	0.80	0.36	0.73	
Zhengzhou	0.82	0.32	0.72	
Hefei	0.81	0.31	0.71	
Tianjin	0.82	0.29	0.70	
Xiamen	0.82	0.30	0.70	
Qingdao	0.81	0.28	0.69	Primary coordination
Jinan	0.76	0.29	0.69	
Changsha	0.72	0.30	0.68	
Fuzhou	0.74	0.31	0.69	
Kunming	0.77	0.25	0.66	
Guiyang	0.82	0.23	0.66	
Nanning	0.81	0.23	0.66	
Shenyang	0.79	0.23	0.65	
Taiyuan	0.82	0.22	0.65	
Nanchang	0.81	0.22	0.65	
Dalian	0.78	0.22	0.64	
Lanzhou	0.82	0.21	0.64	
Haikou	0.78	0.22	0.64	
Changchun	0.81	0.20	0.63	
Harbin	0.80	0.19	0.62	
Hohhot	0.81	0.18	0.62	
Urumqi	0.82	0.17	0.61	
Yinchuan	0.81	0.17	0.61	
Shijiazhuang	0.70	0.19	0.60	
Xining	0.81	0.16	0.60	

**Table 5:** The influencing factor of the level of coupling coordination.

Influencing Factors	Influencing Factors	Influencing Factors	Unit
Economic level	X1	GDP per capita ^1^	Yuan/person
Industry structure	X2	Value added by the tertiary industry as a proportion of GDP ^1^	%
Government regulation	X3	General public budget expenditure per capita ^1^	Million yuan
Traffic condition	X4	Urban road area per capita ^1^	Square meters/person
Population size	X5	Number of residents ^1^	10,000 people

^1^ Data source: the 2021 China Urban Statistical Yearbook (accessed on 26 June 2022).

**Table 6:** Results of dominant factor detection.

Indicators	X1	X2	X3	X4	X5
q	0.48 ***	0.14 ***	0.52 ***	0.33 ***	0.53 ***
Rank	3	5	2	4	1

*** *p* < 0.01; q indicates the influence coefficient of each detection factor.

**Table 7:** Results of dual-factor detection.

Interaction Factors	q (M∩N)	q (M)	q (N)	Interaction Results
X1 ∩ X2	0.87	0.48	0.14	Linear enhancement
X1 ∩ X3	0.78	0.48	0.52	Dual-factor enhancement
X1 ∩ X4	0.86	0.48	0.33	Linear enhancement
X1 ∩ X5	0.88	0.48	0.70	Dual-factor enhancement
X2 ∩ X3	0.86	0.14	0.66	Linear enhancement
X2 ∩ X4	0.94	0.14	0.33	Linear enhancement
X2 ∩ X5	0.86	0.14	0. 53	Linear enhancement
X3 ∩ X4	0.81	0.52	0.33	Dual-factor enhancement
X3 ∩ X5	0.93	0.52	0. 53	Dual-factor enhancement
X4 ∩ X5	0.91	0. 33	0. 53	Linear enhancement

## Data Availability

Not applicable.
